# 17β-estradiol regulates the expression of apolipoprotein M through estrogen receptor α-specific binding motif in its promoter

**DOI:** 10.1186/s12944-017-0458-x

**Published:** 2017-03-31

**Authors:** Jiang Wei, Yang Yu, Guang-hua Luo, Yue-hua Feng, Yuan-ping Shi, Jun Zhang, Qin-feng Mu, Miao-mei Yu, Li-li Pan, Maria Berggren-Söderlund, Peter Nilsson-Ehle, Xiao-ying Zhang, Ning Xu

**Affiliations:** 1 Department of Comprehensive Laboratory, the Third Affiliated Hospital of Soochow University, Changzhou, 213003 China; 2 Cardiothoracic Surgery, the Third Affiliated Hospital of Soochow University, Changzhou, 213003 China; 3 Division of Clinical Chemistry and Pharmacology, Department of Laboratory Medicine, Lunds University, S-221 85 Lund, Sweden

**Keywords:** Estrogen receptor alpha, Apolipoprotein M, Estrogen responsive element, Electrophoretic mobility shift assay (EMSA), Chromatin Immunoprecipitation (ChIP) assay

## Abstract

**Background:**

We have previously demonstrated that estrogen could significantly enhance expression of apolipoprotein M (apoM), whereas the molecular basis of its mechanism is not fully elucidated yet. To further investigate the mechanism behind the estrogen induced up-regulation of apoM expression.

**Results:**

Our results demonstrated either free 17β-estradiol (E2) or membrane-impermeable bovine serum albumin-conjugated E2 (E2-BSA) could modulate human apoM gene expression via the estrogen receptor alpha (ER-α) pathway in the HepG2 cells. Moreover, experiments with the luciferase activity analysis of truncated apoM promoters could demonstrate that a regulatory region (from-1580 to −1575 bp (−GGTCA-)) upstream of the transcriptional start site of apoM gene was essential for the basal transcriptional activity that regulated by the ER-α. With the applications of an electrophoresis mobility shift assay and a chromatin immunoprecipitation assay, we could successfully identify a specific ER-α binding element in the apoM promoter region.

**Conculsion:**

In summary, the present study indicates that 17β-estradiol induced up-regulation of apoM in HepG2 cells is through an ER-α-dependent pathway involving ER-α binding element in the promoter of the apoM gene.

## Background

Apolipoprotein M (apoM) is mainly located in high density lipoprotein (HDL) in human plasma, with only a small proportion presented in low density lipoprotein (LDL) and very low density lipoprotein (VLDL) particles [1]. In humans and mice, apoM is mainly expressed in hepatocytes and in kidney proximal tubule epithelial cells [2, 3]. The apoM-containing lipoproteins were predominantly of HDL size, and about 5% of the total HDL population contained apoM in human plasma [4]. Wolfrum and his colleagues, by using apoM-deficient mice, demonstrated that apoM is important for preβ-HDL formation and cholesterol efflux from macrophages; thus, apoM-deficient HDL was markedly less efficient in facilitating cholesterol efflux from macrophages in vitro than normal HDL [5]. Moreover, over-expression of apoM in LDL-receptor knock-out mice protected against atherosclerosis when fed a high cholesterol diet [5]. Recently, apoM was shown to be a carrier for sphingosine 1-phosphate (S1P), a bioactive lipid mediator. HDL-associated S1P is bound specifically to both human and murine apoM. Isolated human apoM^+^ HDL contained S1P, whereas apoM^−^HDL did not. Moreover, HDL in apoM^−/−^mice contains no S1P, whereas HDL in transgenic mice overexpressing human apoM has an increased S1P content [6]. In another study it showed that hepatic overexpression of apoM could stimulate formation of larger apoM/sphingosine 1-phosphate-enriched high density lipoprotein in plasma [7]. It is well known that S1P is a critical regulator of many physiological and pathophysiological processes, including cancer, atherosclerosis, diabetes and osteoporosis [8].

We have previously demonstrated that estrogen could significantly enhance the expression of apoM in dose-dependent and time-dependent manner, whereas the molecular basis of its mechanism is unknown yet [9]. In general the action of estrogen can be mediated by the classic nuclear estrogen receptors (ER), ER-αand ER-β or through membrane receptors. Mechanisms by which ER-α and ER-β bind ligand, dimerize, associate with enhancers or corepressors, and regulate gene transcription through binding to target genes, are well known and are typically referred to as genomic actions. In addition, membrane associated ERs mediate nongenomic actions of estrogens. These responses occur rapidly, within seconds to few minute. ER-α is one of estrogen receptor family. It is mainly expressed in reproductive tissues, kidney, bone, white adipose tissues and liver, and localized in nucleus, plasma member and mitochondria [10, 11]. Haas et al. examined the effects of isoflavones (quercetin, isoquercetin, and myricetin) on apoA-I gene expression in HepG2 and Caco-2 cells. They reported that quercetin could upregulate apoA-I gene expression at least in part by ER-α, which might be applicable for the treatment of hypoalphalipoproteinemia [12]. As a major component of HDL, apoA-I help to clear fats, including cholesterol, from white blood cells within artery walls, making the white blood cells less likely to become fat overloaded, transform into foam cells, die and contribute to progressive atheroma. Moreover, estrogen stimulates expression of chicken hepatic vitellogenin II and very low density apolipoprotein II through ER-α [13].

## Methods

### Materials

The HepG_2_ and MCF-7cell lines were obtained from the American Type Culture Collection (Manassas, VA, USA). Six-well cell culture clusters and 75 cm^2^ vented cell culture flasks were purchased from Nunc (Roskilde, Denmark). 17β-estradiol (E2) and E2 Conjugated with Bovine Serum Albumin (E2-BSA, membrane impermeable E2) from sigma chemical Co.Ltd. (Shanghai, China). Fetal bovine serum (FBS), DMEM, phenol red-free DMEM and charcoal treated FBS were obtained from Wissent (Nanjing, China).1,3-Bis(4-hydroxyphenyl)-4-methyl-5-[4–2-piperidinyletho- xy)phenol]-1H–pyrazole (MPP) dihydrochloride was purchased from Tocris bioscience Co. Ltd. (Shanghai, China). Rabbit anti-human apoM monoclonal antibodies were from Abnova Corporation (Taiwan, china). Total RNA purification kits were purchased from the Shenergy Biocolor BioScience and Technology Company (Shanghai, China). First strand cDNA synthesis kitswere obtained from Fermantas (Vilnius, Lithuania). The LightCycler real-time RT-PCR System was from Roche Applied Science (Mannheim, Germany).

### Cell cultures

HepG2 and MCF-7 cells were cultured in DMEM supplemented with 10% FBS in the presence of 100 U/ml penicillin, 100 μg/ml streptomycin and 1% Glutamax at 37 °C under 5% CO_2_ atmosphere. HepG2 cells were plated in 6-well cell culture clusters at a density of 5 × 10^4^ cells/dish with phenol red–free DMEM containing 10% charcoal-treated FBS. Cell monolayers of approximately 50–70% confluence were grown for 24 h in the above media, then washed and incubated in serum-free medium with different concentrations of of E2-BSA(1 ~ 100 nM) for 24 h, before extraction of total RNA. In the antagonism study, we used E2 (10 μM) or E2-BSA (100 nM) and MPP (1 μM) in the culture medium. E2 and MPP were dissolved in ethanol, E2-BSA was dissolved in DMEM.

### Total RNA extraction and real time RT-PCR

Total RNA extraction and real time RT-PCR were referred as our previous methods [9]. In brief, quantifications of apoM mRNA levels are relative to mRNA level of GAPDH, and were performed on a LightCycler in a final volume of 25 μl. Optimum reaction conditions were obtained with 2.5 μl of 10 × PCR buffer, 1.5 μl of 25 mM MgCl_2_, 0.5 μl of 10 mM 4 × dNTPs, 0.25 μl of 5 U/μl common Taq DNA polymerase, 0.1 μl of 100 μM specific sense primer(s), 0.1 μl of 100 μM specific antisense primer(s), 0.1 μl of 100 μM specific probe(s) and 2 μl template cDNA. Finally 17.95 μl ddH_2_O was added to the reaction mixture. The thermal cycling conditions for GAPDH and apoM included the following steps: 25 °C for 10 min, 48 °C for 30 min and 95 °C for 5 min to do reverse transcription, and then the reaction mixture was preheated for for 3 min at 95 °C. After that, a 40-cycle two-step PCR was performed consisting of 5 s at 95 °C and 27 s at 58 °C. Samples were amplified simultaneously in triplicates in one-assay run.

### Western blot analysis

ApoM concentrations in cells were measured by western blot analysis [9]. In brief, for cell extract preparation, cells were resuspended in RIPA lysis buffer. Aliquots of cell extracts were resolved on a 10% SDS-polyacrylamide gel electrophoresis, transferred electrophoretically to nitrocellulose membranes, and the primary antibodies used were apoM and GAPDH. Horseradish peroxidase-conjugated second antibody was used in conjunction with ECL chemiluminescence detection system (Amersham) and quantified by a scanner using the Quqntity One software (Version 4.2.1, Bio-Rad Laboratories).

### Construction of the human apoM activated luciferase (hapoM-luc) reporter plasmids

Genome DNA was isolated from the MCF-7 cells using a DNA extraction kit (QIAGEN) and dissolved in water. Based upon our findings regarding the location of the transcriptional start site, hapoM-luc constructed with the human apoM promoter sequences (−3060/0 (P1), −2207/0 (P2), −1740/0 (P3) and −1189/0 (P4)) were generated by PCR amplification of corresponding fragments, and subsequent cloning into Bgl II-Hind III sites of the pMD-18 T Vetor (TaKaRa Corp). The mutation of hapoM-luc which bears mutations in the ER-α binding element was generated by site-directed mutagenesis using the QiuckChange Site-Directed mutagenesis Kit (Stratagene) according to the manufacturer’s instructions. A 2880-bp fragment from 2828 to **+**52 in the 5'-flanking region of the USP22 gene was generated by PCR. The amplified DNA was cloned into pMD 18 T simple vector and verified by direct sequencing. Other deletion fragments were generated by PCR using this plasmid DNA as a template (see Table 1 for PCR primer sequences). The PCR products were gel-purified, digested with KpnI and BglII, and subcloned into the pGL3-basic firefly luciferase vector (Promega). All the sequences of the cloned promoter region were confirmed by DNA sequencing. These sequences of the USP22 promoter were analyzed for the presence of consensus transcription factor binding sites using the Mat Inspector program (Genomatix Software GmbH). Site-directed mutagenesis to inactivate the ER-α binding sites at positions −1580 to −1575 of the promoter were carried out within the p-210/**+**52 construct according to the MutanBEST Kit methodology (TaKaRa Bio) with the following primers: mutant forward primer, 5′-TTA ATA AAC TCT AAT ATA CTC ACT GCC CAA ATT TTG TTT GTT TTT-3′;mutant reverse primer, 5′-AAA AAC AAA CAA AAT TTG GGC AGT GAG TAT ATT AGA GTT TAT TAA-3′. All mutations were confirmed by DNA sequencing.

**Table 1:** Primers

Name	Sequence 5′- 3′	Enzyme site
apoM P1 F	ggaAGATCTTCTTGGCAGGCTGATAATTTCAGG	BglII
apoM P2 F	ggaAGATCTTCACAACTCACTGTAGCCTCTGC	BglII
apoM P3 F	ggaAGATCTCAGCTTGGGCAACAGAACG	BglII
apoM P4 F	ggaAGATCTTACCCACCAGAAACTAAGTG	BglII
apoM P R	ttacccAAGCTTGGAGCTGGTGCTCTGTGTGCC	Hind III

### Cloning of ER-α expression plasmid

Primers were designed with incorporation of Xho I and EcoRI restriction site. These primers used in the subsequence PCR amplification of ER-α were: forward primer, 5′- tac gtc CTC GAG ATG ACC ATG ACC CTC CAC ACC A-3′, and reverse primer, 5′-ccg GAA TTC TCA GAC CGT GGC AGG GAA AC-3′. The PCR products were confirmed by DNA sequencing and digested with Xho I and EcoRI and inserted into pCDNA3.1.

### Transfections and dual luciferase reporter assay

MCF-7 cells were plated in 24-well plates for 24 h before transfection with 0.5 mg of various apoM promoter constructs and 0.1 mg of pRL-TK (Promega) using Lipofectamine 2000 (Invitrogen) in each well. All transfection experiments were repeated five times. Twenty-four hours after transfection, cells were washed in phosphate-buffered saline and lysed for 30 min at room temperature using passive lysis buffer (Promega). Luciferase activity was determined using the dual activity was expressed as the ratio of firefly luciferase activity to Renilla luciferase for each sample.

### Electrophoretic mobility shift assay (EMSA)

Nuclear protein extract was isolated with Nucleoprotein Extraction Kit (Beyotime). Double-stranded oligonucleotides (Sangon) corresponding to the ER-α binding sites of the apoM promoter were synthesized and annealed into double strands. The DNA binding activity of ER-α protein was detected by LightShift® Chemiluminescent EMSA Kit (Pierce). 5 μg nuclear protein extract was added to 1 nmol Biotin-labeled double stranded oligonucleotides, 1 × Binding Buffer, 2.5% Glycerol, 5 mM MgCl_2_, 50 ng Poly (dI·dC), 0.1 mM EDTA and 0.05% NP-40. In addition, control group was added 2 pmol unlabeled competitor oligonucleotides, while the super-shift group was added 10 μg ER-α antibody (Santa Cruz). The mixtures were then incubated at 4 °Cfor 20 min. The reactions were analyzed by electrophoresis in 6.5% polyacrylamide gels at 100 V for 1 h, and then transferred to a nylon membrane. The dried nylon was scanned with GE ImageQuant LAS4000 mini (GE-Healthcare).

### Chromatin Immunoprecipitation (ChIP) assay

ChIP assays were performed according to the Piece Agarose Chip Kit (piercenet) manufacturer instructions. Briefly, MCF-7 cells were fixed by adding formaldehyde to a final concentration of 1% and incubated by modest shaking for 10 min at room temperature. Thereafter, cells were washed twice with cold phosphate-buffered saline. The pellet was resuspended and lysed, and nuclei were isolated and sonicated until the chromatin had an average length of 500–1500 bp. After centrifugation, the supernatant was incubated with 3 mg of antibody against ER-α overnight at 4 °C for immunoprecipitation. The following day, Agrose resin was added and the mix was further incubated at 4 °C for 1 h. After appropriate washing, the antibody-transcription factor-DNA complex was eluted, formaldehyde cross-links were reversed, and proteins were digested with proteinase K at 65 °C 1.5 h. DNA was purified and used for PCR with primers 5’TTG GGC AAC AGA ACG AGA CT 3′ and 5’TAT CCC ATG GAC TGC CAC AA3’. The product sequence is between −1676 and −1519 bp of apoM promoter.

### Statistical analysis

A Prism 6.0 software package (GraphPad Software, Inc., CA, USA) was utilized for statistical analysis. The data were compared by ANOVA and followed by Bonferroni’s method for multiple comparisons. Significance level was set at *P*<0.05.

## Results

### Estrogen induced up-regulation of apoM expression is via the ER-α

**Fig. 1:**
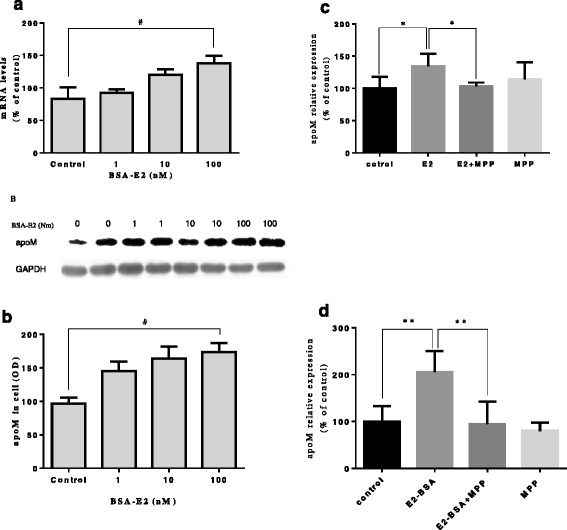
Effects of estrogens and MPP on the apoM levels of in HepG2 cells. HepG2 cells were cultured with E2-BSA (1 ~ 100 nM) for 24 h (**a** and **b**). HepG2 cells were cultured with E2 (10 μM) or E2-BSA (100 nM) together MPP (1 μM) for 24 h (**c** and **d**). The mRNA and protein levels of apoM were determined as described in the materials and methods. Data are means ± SE. The control groups are represented as 100%. ^#^ *P* < 0.05 vs control; ^*^ *P* < 0.05 vs E2;^**^ *P <* 0.01 vs. E2 + BSA

As shown in Fig. 1a and Fig. 1b, 100 nM BSA-E2 significantly upregulated mRNA and protein levels of apoM (*p* < 0.05). The stimulatory effects of BSA-E2 on mRNA and protein levels of apoM in the HepG2 cells were dose-dependent manner. The ER-α antagonist, MPP, could abolish the estrogens-induced up-regulation of apoM (*P* < 0.05), whereas MPP itself did not significantly influence mRNA levels of apoM in HepG_2_ cell cultures (*P* > 0.05) (Fig. 1c and Fig. 1d).

### Identification of ER-α binding site of the human apoM promoter

**Fig. 2:**
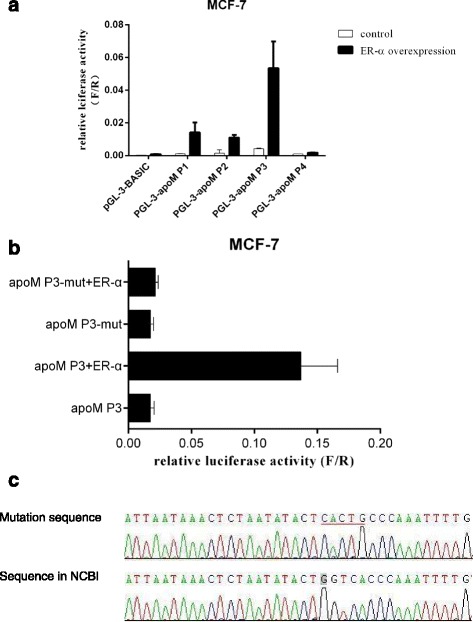
Characterization of the ER-α response sequence in the apoM gene promoter. **a** Luciferase reporter assay for the apoM promoter. A series of truncated apoM promoter-reporter constructs were schematized. Each promoter-reporter construct or the promoter-less plasmid pGL3-basic was co-transfected with ER-α expression plasmid into MCF-7 cells. After 24 h of transfection, the cells were lysed and measured for firefly and renilla luciferase activities. The luciferase activity of each construct was presented relative to the pGL3-basic activity. **b** Mutation analysis of the ER-α binding site. Luciferase activity expressed by the ER-α site-directed mutant relative to pGL3-basic activity. **c** Compared with the existing information in human genome database, the DNA sequence of the mutation contains a mutation region, from −1580 to −1575 upstream from the transcriptional start site

As described in the materials and methods, various truncated apoM promoter were amplified and cloned into the luciferase reporter vector pMD-18 T Basic and then were transfected MCF-7 cells. As shown in Fig. 2a, the P3 (−1740/0 bp), was able to drive expression of the luciferase reporter gene in MCF-7 13 times higher than the pGL3-Basic construct, which indicates the 1740 nucleotides immediately 5' to the transcriptions start site contain the regulatory elements.

Since the region is required for basal transcriptional activity of the apoM gene, we substituted CACTG for the consensus sequence GGTCA (−1580 to −1575 bp) into the parental vector to generate P3 mutant. After the transient transfection, P3 mutant construct showed an approximately 1300% lower luciferase activity in MCF-7 compared with the P3 WT construct, which suggest that this DNA motif plays a positive role toward apoM promoter activity (Fig. 2b).

Compared with the existing information in the human genome database, the DNA sequence of the mutation contains one mutation, GGTCA → CACTG, at position −1580 and −1575 upstream from the transcriptional start site (Fig. 2c).

### Analysis of the binding of the ER to apoM by EMSA

**Fig. 3:**
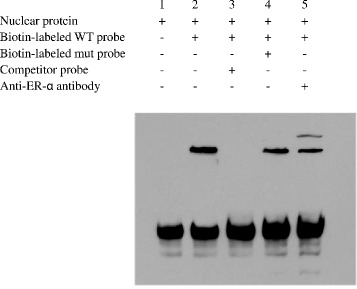
Identification of ER-α responsive element within apoM promoter by EMSA. nuclear extract isolated from MCF-7 cells were incubated overnight with different probes with or without antibody against human ER-α at 4 °C

We next demonstrated the binding of the ER-α to the apoM promoterusing EMSA. In this assay, we used nuclear protein extracts of MCF-7 cells which contained endogenous ER-α. As shown in Fig.3, lane 1 represents the free probe. When incubated with biotin-labeled apoM probe with an MC-7 cell nuclear extract, t a specific protein-DNA complex was formed (Fig.3 lane 2), which was supershifted by anti- ER-α antibody (Fig.3 lane 5). The addition of competitor (an unlabeled apoM probe oligonucleotides) could abrogate the complex formation between the bio-labeled probe and the nuclear extract (Fig.3 lane 3), but the addition of biotin-labeled mutant probe cannot inhibit formation of the specific complexes (Fig.3 lane 4).

**Fig. 4:**
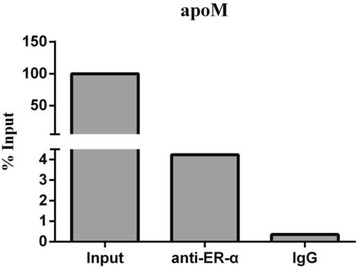
Ch IP-PCR assays with ER-α antibody. Chromatin from MCF-7 cells was immunoprecipitated with anti-ER-α or non-immune IgG (negative control). Input was total chromatin DNA as a positive control, which was analyzed by RT-PCR. The RT-PCR data are normalized to the amount of chromatin in the immunoprecipitated reaction. Data are represented as a ratio of the immunoprecipitated to input DNA

### Chip analysis of the binding of the ER-α to apoM

To demonstrate that some ER-α binding regions located in the apoM promoter in intact cells under in vivo conditions, we carried out a ChIP analysis with anti- ER-α antibody in MCF-7 cells. After chromatin form MCF-7 cells immunoprecipitated with ER-α antibody, we analyzed the recovered chromatin sampled by RT-PCR. Data showed that the apoM expression level of anti-ER-α group was about 12 times that of IgG group (Fig. 4), which implicated the endogenous ER-α directly bind to the apoM promoter (sequence between −1676 and −1519) in MCF-7.

## Discussion

apoM is a human apolipoprotein predominantly located in HDL [1]. Several previous studies showed that some cytokines and nuclear factors regulate apoM expression, including platelet activating factor (PAF), leptin, transforming growth factor (TGF), liver x receptor (LXR) and hepatocyte nuclear factor-1α (HNF-1α) [14–19]. Our previous study found that estrogen increases apoM expression. Thus, it is interesting to determine the regulatory mechanisms that estrogen controls apoM expression. We know that estrogen is a physiologic regulator of blood lipid. However, to date there have been no data regarding the modulation of apoM expression by estrogen. In this study, we used E2 and ER-α antagonist to treat HepG2 cells to determine the effect of estrogen on the expression of apoM and its relative signal pathway. Our results showed that E2 upregulated apoM expression depending on ER-α which belongs to the nuclear receptor family of ligand activated transcription factors. In this study, E2-BSA also induced the transcription of apoM via membrane ER-α, which is likely contrary to this opinion that membrane ER-α mediates rapid non-genomic effects of E2 [20]. But some authors thought membrane ER-α is also involved genes transcription. They developed a point mutant ER-α knockin mouse that precludes palmitoylation and membrane trafficking of the steroid receptor in all organs. mRNAs implicated in organ development were often poorly stimulated by estrogen only in homozygous nuclear-only ER-α mice [21]. To address the molecular mechanisms underlying ER-α increased apoM transcription, we performed promoter truncation together with a luciferase activity assay and site mutation. Results showed that a regulatory region (−GGTCA-,-1580 to −1575 bp) of the apoM promoter was required for apoM basal transcriptional activity, and site mutation abolished the transcriptional activity. The regulatory region (GGTCA) is a half of the consensus estrogen receptor element (ERE) motifs, ERE (GGTCA nnn TGACC) is a 13 bp palindromic inverted repeat with a three nucleotide spacer. Several reports suggest that many estrogen target genes are regulated by diverse elements, such as imperfect EREs and ERE half sites (ERE 1/2), which are either the proximal or the distal half of palindrome [22–24]. Moreover, we further confirmed the formation of apoM-ER-α complex in a protein-DNA style. Some studies have found that there were some nuclear receptors binding regions in apoM promoter [19, 25]. Using base deletion analysis combined with DNA affinity precipitation and chromatin immunoprecipitation assays, venteclef and his colleagues revealed that HNF-4binds to a hormone response element (HRE) in the proximal apoM promoter (nucleotides −33 to −21). The importance of ER-αin lipid metabolism is also supported by some studies which showed a statistical significant association between ER-α and abnormalities in LDL and HDL [26–28]. Figtree and his colleagues found a novel polymorphism (C > T,ERNE-45) upstream of ER-α abolished negative transcriptional regulation by an adjacent glucocorticoid receptor binding sequence, and was strongly associated with HDL levels in a large cohort of post-menopausal woman [28].

In this study, we showed apoM as a novel target gene of hormone nuclear receptor ER-α. Some previous studies found some nuclear receptors regulate apoM expression, including Liver receptor homologue-1 (LRH-1), HNF-4 and LXR [18, 19, 25]. In one of our previous studies, Zhang et al. found that oral administration of the synthetic LXR agonist T09031317 in mice was associated with a down-regulation in basal apoM mRNA levels in the liver. In another study, it is shown that the orphan nuclear receptor liver receptor homolog-1, LRH-1 directly regulates human and mouse apoM transcription by binding to an LRH-1 response element located in the proximal apoM promoter region. In addition, the authors demonstrated that bile acids suppress apoM expression in a SHP-dependent mannaer in vitro and in vivo by inhibiting LRH-1transcriptional activity on the apoM promoter. ApoA-I is also the main structural protein in HDL. Therefore, we speculated that activated of ER-α may contribute to increasing of apoM transcription.

## Conclusions

We have investigated the effects of estrogens on regulation of expression of apoM gene, indicating that estrogens-induced increasing of apoM transcriptional activity is mediated through ER-α binding region in the apoM promoter.

## Abbreviations

ApoM: Apolipoprotein M
ChIP: Chromatin Immunoprecipitation
E2: 17β-estradiol
E2-BSA: Bovine serum albumin conjugated E2
EMSA: Electrophoretic mobility shift assay
ERE: Estrogen receptor element
ER-α: Estrogen receptor alpha
FBS: Fetal bovine serum
HDL: High density lipoprotein
HNF: Hepatocyte nuclear factor
HRE: Hormone response element
LDL: Low density lipoprotein
LRH-1: Liver receptor homologue-1
LXR: Liver x receptor
MPP: 1,3-Bis(4-hydroxyphenyl)-4-methyl-5-[4–2-piperidinylethoxy)phenol]-1H–pyrazole
PAF: Platelet activating factor
S1P: Sphingosine −1-phosphate
TGF: Transforming growth factor
VLDL: Very low density lipoprotein

## References

[1] Xu N, Dahlback B (1999). A novel human apolipoprotein (apoM). J Biol Chem.

[2] Zhang XY, Dong X, Zheng L, Luo GH, Liu YH, Ekstrom U, Nilsson-Ehle P, Ye Q, Xu N (2003). Specific tissue expression and cellular localization of human apolipoprotein M as determined by in situ hybridization. Acta Histochem.

[3] Zhang XY, Jiao GQ, Hurtig M, Dong X, Zheng L, Luo GH, Nilsson-Ehle P, Ye Q, Xu N (2004). Expression pattern of apolipoprotein M during mouse and human embryogenesis. Acta Histochem.

[4] Christoffersen C, Nielsen LB, Axler O, Andersson A, Johnsen AH, Dahlback B (2006). Isolation and characterization of human apolipoprotein M-containing lipoproteins. J Lipid Res.

[5] Wolfrum C, Poy MN, Stoffel M (2005). Apolipoprotein M is required for prebeta-HDL formation and cholesterol efflux to HDL and protects against atherosclerosis. Nat Med.

[6] Karuna R, Park R, Othman A, Holleboom AG, Motazacker MM, Sutter I, Kuivenhoven JA, Rohrer L, Matile H, Hornemann T (2011). Plasma levels of sphingosine-1-phosphate and apolipoprotein M in patients with monogenic disorders of HDL metabolism. Atherosclerosis.

[7] Liu M, Seo J, Allegood J, Bi X, Zhu X, Boudyguina E, Gebre AK, Avni D, Shah D, Sorci-Thomas MG (2014). Hepatic apolipoprotein M (apoM) overexpression stimulates formation of larger apoM/sphingosine 1-phosphate-enriched plasma high density lipoprotein. J Biol Chem.

[8] Maceyka M, Harikumar KB, Milstien S, Spiegel S (2012). Sphingosine-1-phosphate signaling and its role in disease. Trends Cell Biol.

[9] Wei J, Shi Y, Zhang X, Feng Y, Luo G, Zhang J, Mu Q, Tang Y, Yu Y, Pan L (2011). Estrogen upregulates hepatic apolipoprotein M expression via the estrogen receptor. Biochim Biophys Acta.

[10] Matthews J, Gustafsson JA (2003). Estrogen signaling: a subtle balance between ER alpha and ER beta. Mol Interv.

[11] Merchenthaler I, Lane MV, Numan S, Dellovade TL (2004). Distribution of estrogen receptor alpha and beta in the mouse central nervous system: in vivo autoradiographic and immunocytochemical analyses. J Comp Neurol.

[12] Haas MJ, Onstead-Haas LM, Szafran-Swietlik A, Kojanian H, Davis T, Armstrong P, Wong NC, Mooradian AD (2014). Induction of hepatic apolipoprotein A-I gene expression by the isoflavones quercetin and isoquercetrin. Life Sci.

[13] Li J, Leghari IH, He B, Zeng W, Mi Y, Zhang C (2014). Estrogen stimulates expression of chicken hepatic vitellogenin II and very low-density apolipoprotein II through ER-alpha. Theriogenology.

[14] Xu N, Zhang XY, Dong X, Ekstrom U, Ye Q, Nilsson-Ehle P (2002). Effects of platelet-activating factor, tumor necrosis factor, and interleukin-1alpha on the expression of apolipoprotein M in HepG2 cells. Biochem Biophys Res Commun.

[15] Xu N, Nilsson-Ehle P, Hurtig M, Ahren B (2004). Both leptin and leptin-receptor are essential for apolipoprotein M expression in vivo. Biochem Biophys Res Commun.

[16] Luo G, Hurtig M, Zhang X, Nilsson-Ehle P, Xu N (2005). Leptin inhibits apolipoprotein M transcription and secretion in human hepatoma cell line, HepG2 cells. Biochim Biophys Acta.

[17] Xu N, Hurtig M, Zhang XY, Ye Q, Nilsson-Ehle P (2004). Transforming growth factor-beta down-regulates apolipoprotein M in HepG2 cells. Biochim Biophys Acta.

[18] Zhang X, Zhu Z, Luo G, Zheng L, Nilsson-Ehle P, Xu N (2008). Liver X receptor agonist downregulates hepatic apoM expression in vivo and in vitro. Biochem Biophys Res Commun.

[19] Mosialou I, Zannis VI, Kardassis D (2010). Regulation of human apolipoprotein m gene expression by orphan and ligand-dependent nuclear receptors. J Biol Chem.

[20] Campbell CH, Bulayeva N, Brown DB, Gametchu B, Watson CS (2002). Regulation of the membrane estrogen receptor-alpha: role of cell density, serum, cell passage number, and estradiol. FASEB J.

[21] Pedram A, Razandi M, Lewis M, Hammes S, Levin ER (2014). Membrane-localized estrogen receptor alpha is required for normal organ development and function. Dev Cell.

[22] Petz LN, Ziegler YS, Loven MA, Nardulli AM (2002). Estrogen receptor alpha and activating protein-1 mediate estrogen responsiveness of the progesterone receptor gene in MCF-7 breast cancer cells. Endocrinology.

[23] Menuet A, Le Page Y, Torres O, Kern L, Kah O, Pakdel F (2004). Analysis of the estrogen regulation of the zebrafish estrogen receptor (ER) reveals distinct effects of ERalpha, ERbeta1 and ERbeta2. J Mol Endocrinol.

[24] Vyhlidal C, Samudio I, Kladde MP, Safe S (2000). Transcriptional activation of transforming growth factor alpha by estradiol: requirement for both a GC-rich site and an estrogen response element half-site. J Mol Endocrinol.

[25] Venteclef N, Haroniti A, Tousaint JJ, Talianidis I, Delerive P (2008). Regulation of anti-atherogenic apolipoprotein M gene expression by the orphan nuclear receptor LRH-1. J Biol Chem.

[26] Jones DR, Schmidt RJ, Pickard RT, Foxworthy PS, Eacho PI (2002). Estrogen receptor-mediated repression of human hepatic lipase gene transcription. J Lipid Res.

[27] Kajinami K, Brousseau ME, Lamon-Fava S, Ordovas JM, Schaefer EJ (2005). Gender-specific effects of estrogen receptor alpha gene haplotype on high-density lipoprotein cholesterol response to atorvastatin: interaction with apolipoprotein AI gene polymorphism. Atherosclerosis.

[28] Figtree GA, Grieve SM, Speller B, Geiger MJ, Robinson BG, Channon KM, Ragoussis J, Collins P, Watkins H (2008). A commonly occurring polymorphism upstream of the estrogen receptor alpha alters transcription and is associated with increased HDL. Atherosclerosis.

